# HLA-B27 Modulates Intracellular Growth of *Salmonella* Pathogenicity Island 2 Mutants and Production of Cytokines in Infected Monocytic U937 Cells

**DOI:** 10.1371/journal.pone.0034093

**Published:** 2012-03-28

**Authors:** Shichao Ge, Qiushui He, Kaisa Granfors

**Affiliations:** 1 Department of Infectious Disease Surveillance and Control, National Institute for Health and Welfare, Turku, Finland; 2 Department of Pediatrics, University of Turku, Turku, Finland; University of Leuven, Rega Institute, Belgium

## Abstract

**Background:**

*Salmonella enterica* serovar Enteritidis PT4 KS8822/88 replicates rapidly in HLA-B27-transfected human monocytic U937 cells. In this process, *Salmonella* pathogenicity island 2 (SPI-2) genes play a crucial role. Our previous study indicated that 118 *Salmonella* genes, including 8 SPI-2 genes were affected by HLA-B27 antigen during *Salmonella* infection of U937 cells.

**Methods/Principal Findings:**

To further investigate *Salmonella* replication in HLA-B27-positive U937 monocytic cells, two SPI-2 genes, *ssaS* and *sscA* up-regulated most during *Salmonella* infection of HLA-B27-transfected U937 cells, were mutated by using one-step gene disruption method. Intracellular survival and replication of the mutants in the U937 cells was compared to that of the wild type strain. Surprisingly, the two mutated strains replicated significantly more than the wild type bacteria in HLA-B27-transfected cells. Secretion of tumor necrosis factor alpha (TNF-α) and interleukin 10 (IL-10) was significantly induced during the infection of HLA-B27-transfected U937 cells with the mutants. The results indicated that the certain SPI-2 genes in wild type bacteria suppress *Salmonella* intracellular growth and production of cytokines in infected HLA-B27-transfected cells. HLA-B27-associated modulation of *Salmonella* SPI-2 genes and cytokine production may have importance in the persistent infection of the bacteria and the pathogenesis of reactive arthritis.

**Conclusions:**

The study provides evidence that certain virulence factors of pathogens can reduce the intracellular growth in the host cells. We suggest that the limiting intracellular growth might be a strategy for persistence of bacteria in host cells, keeping a balance between pathogenic growth and pathogenesis.

## Introduction

Reactive arthritis (ReA) is a joint inflammation that occurs following infections with certain intracellular Gram-negative bacteria, e g. *Salmonella* and *Yersinia* [1]. The development and severity of ReA is strongly associated with the human leukocyte antigen-B27 (HLA-B27) [2], [3]. However, the mechanism by which HLA-B27 confers disease susceptibility remains elusive. Nevertheless, a consistent feature of most hypotheses suggest that the interaction between the ReA-triggering bacteria and the HLA-B27-positive subjects, in whom ReA often develops, is abnormal and leads to an inefficient elimination of the causative bacteria [4], [5]. Our previous studies demonstrated impaired elimination [6]–[8] or enhanced replication [9]–[11] of *Salmonella* Enteritidis in HLA-B27-transfected human monocytic cells, as compared with appropriate controls expressing other HLA class I antigens. However, it has been less known how HLA-B27 regulates bacterial intracellular growth in the molecular level.

Monocytes/macrophages are important cells harboring pathogens causing persistent infections or systemic disease in the body, but in order to survive and replicate the bacteria need to resist the bactericidal responses from the host cells [12]. Secretion of cytokines in host cells is closely associated with the persistence and pathogenesis of bacteria. Tumor necrosis factor alpha (TNF-α) is a pro-inflammatory cytokine and considered one of important cytokines in the development of spondyloarthropathy (SpA). Anti-TNF-α therapies have been proven to be an effective in the treatment of patients suffering from SpA [13], [14]. TNF-α is involved in several physiological and pathological responses of host during bacterial infection [15], [16]. It is associated with host resistance phenotype, activates the bactericidal activity of macrophages [17], [18], and contributes to clearance of *Salmonella* in animal model [19]. While TNF-α was involved in the activation of macrophages, anti-inflammatory cytokines, such as interleukin-10 (IL-10) can have antagonistic effects. IL-10 has been shown to have multiple biological activities, e.g., its major function appears to be suppression of T helper type 1 (Th1) cytokine synthesis including TNF-α and IFN-γ. It also inhibits the host defence by deactivated macrophages [20]. IL-10-treatment caused host cells to become permissive for growth of intracellular pathogens [21]. Activated monocytes and macrophages are the major source of IL-10 production.

The virulence genes in *Salmonella* responsible for infection and pathogenicity are mainly clustered in distinct 40 kb chromosomal region called *Salmonella* pathogenicity islands (SPI), which have been acquired by horizontal gene transfer [22], [23]. SPI-1 and SPI-2 are known as pathogenicity islands, which encode their own type III secretion systems (T3SS) to export bacterial effector proteins into the host [24], [25]. The SPI-1 secretion system mediates bacterial invasion into host intestinal epithelial cells [26] while the SPI-2 is required for *Salmonella* replication inside host cells and systemic infections in mice [27]. The SPI-2 genes were specifically induced inside host cells selected by fluorescence-activated cell sorting [28]. Signals specific to the intracellular environment are sensed and modulated by a two-component regulatory system *ssrA/B* encoded in the SPI-2 region, leading to induction of SPI-2 gene expression [29]. Further study revealed that the expression of *ssrA/B* itself is regulated by OmpR-EnvZ, another two-component system with global regulatory function located outside the SPI-2 region [30]. Moreover, the SPI-2 region contains large blocks of horizontally acquired genes which has a higher A+T content than other parts of *Salmonella* DNA [24], [31]. The AT-rich DNA is bound by the histone-like nucleoid structuring protein (H-NS), leading to the silencing of the binding genes [32]. The H-NS-promoted silencing might protect *Salmonella* from the detrimental effect of over-expressing of SPI-2 virulence genes at inappropriate times [33].

Our recent study indicated that the expression of HLA-B27 in monocytic cells influenced global *Salmonella* gene response during intracellular infection [11]. Expression of *Salmonella* genes was significantly changed during infection of HLA-B27-transfected cells compared to the infection of HLA-A2-transfected cells. SPI-2 genes were the most up-regulated. To learn more about the interplay between the SPI-2 genes and HLA-B27 antigen, two of the SPI-2 genes were mutated and intracellular growth of the mutants was investigated during the course of infection. In addition, the production of cytokines TNF-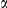 and IL-10 was also determined in *Salmonella* infected cells.

## Results

### Cell surface expression of HLA-B27 and HLA-A2 molecules

The expression of transfected HLA-B27 and HLA-A2 was confirmed by flow cytometry always in new batches of the cells as before [10], [11]. The level of expression of the transfected molecules in U937 cells was similar to that of HLA-B51, one of the MHC class I molecules endogenously expressed by U937 cells. In addition, the surface expression levels of the transfected molecules corresponded to the levels of those molecules endogeneously expressed on peripheral blood monocytes [6].

### Construction of *Salmonella* SPI-2 mutants

**Figure 1:**
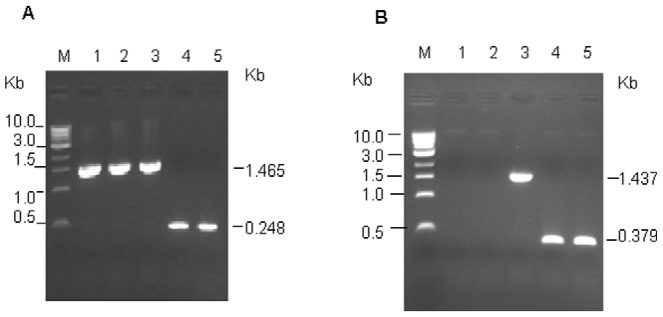
The corresponding DNA PCR product in mutated and wild type genes. The PCR product from three mutated isolates *ssaS*::Km (lanes 1–3) and the wild type gene *ssaS* (lanes 4 and 5 (A), and one mutated isolate *sscA*::Km (lane 3) and wild type gene *sscA* (lanes 4 and 5) (B) are demonstrated. The genomic DNAs isolated from the mutant and wild type bacteria were used as templates, respectively, and the flanking primers (Table 1) used in the PCR reaction.

To further study the intracellular growth of *Salmonella* in HLA-B27-positive U937 monocytic cells, two SPI-2 genes *ssaS* and *sscA*, significantly increased their expression in HLA-B27-transfected cells, were disrupted. Within the internal region of the gene *ssaS* in wild type *S.* Enteritidis PT4 KS8822/88, 33 nucleotides were replaced with the kanamycin gene cassette, and within the internal region of the gene *sscA*, total 72 nucleotides were replaced with the kanamycin gene cassette. Candidate mutant colonies were selected on LB plates with antibiotic kanamycin because the wild type bacteria failed to grow on these plates. Further confirmation was performed by PCR using the flanking primers (Table 1), external to the site of mutation in disrupted genes. The genomic DNA isolated from the wild type and mutant strains respectively were used as templates, and the PCR products with expected sizes were produced (Fig. 1). This indicates that the homologous recombination occurred in the mutated strains and the expected mutants were obtained.

**Table 1:** Primers used in the gene mutagenesis in this study.

*Genes*	*Primers*	*Sequences*
*ssaS*	ssaS-Km-F	5′-CAATTTGTAACGCAACTTTTATGGATCGTCCTTTTTACGTCTATGCCGGTAGTGTTGGTGAAAGCCACGTTGTGTCTCAA-3′ α
	ssaS-Km-R	5′-AATTGCCAATAATTTAATCATGAACTGTAGCGTTTGGTCCTGTATTTGAGTCAAGGCCTGGCGCTGAGGTCTGCCTCGTG-3′ α
	ssaS-F	5′-GACGCAATTTGTAACGCAAC-3′
	ssaS-R	5′-AACCATGCTCTCCAATTCGT-3′
*sscA*	sscA-Km-F	5′-ATATATGATGCCTGGTCATTTGACTACTGGTTTCGGTTAGGGGAATGCTGCCAGGCTCAAAAAGCCACGTTGTGTCTCAA-3′ α
	ssaS-Km-R	5′-TAACGCTTTGATTGCATAACAGACGTTATCACACGCGAGATAGCATTCCGCTGCGGCCCAGCGCTGAGGTCTGCCTCGTG-3′ α
	sscA-F	5′-GCGTATGTTGTTGGATGACG-3′
	sscA-R	5′-CATCTTTTCTGCACGATGTC-3′

α : 80-nucleotide (nt)-long primers including 60 nt homology extensions complementary to the targeted regions of the genes and 20 nt priming sequences (underlined) for the synthesis of the kanamycin resistance cassette gene from *E. coli* MC4100 *ybeW*::Km.

### Growth of mutant and wild type strains in LB broth

**Figure 2:**
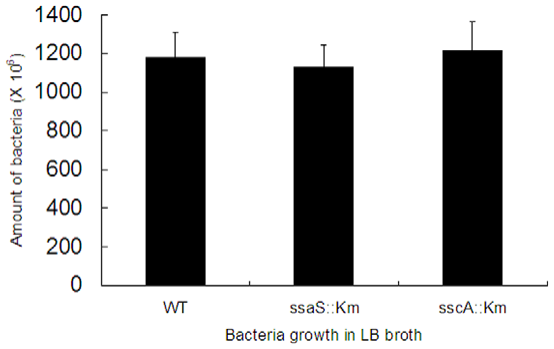
The growth of the mutants and wild type bacteria in LB medium. The mutants and wild type bacteria were grown in LB broth with addition of kanamycin (50 µg/ml) or without it, respectively. The number of bacteria was calculated and reported as CFU (colony forming units), The results represent mean numbers ± standard deviations from three independent experiments with duplicate samples. The results suggested that these mutants and wild type *Salmonella* had a similar growth rate in LB broth.

To investigate whether the mutations on the SPI2 genes affected the growth of the bacteria *in vitro* in LB medium, the mutants and wild type strain were grown as routinely in LB broth overnight. The bacteria cultures were then transferred into fresh LB broth to get a logarithmic growth phase of bacteria for each of them. The bacteria cultures were further diluted and put on LB plates, supplemented with 30 µg/ml of kanamycin for mutant strains to grow. The number of bacteria was calculated, reported as CFU, and the results were shown in Fig. 2. The results suggested that these mutants and wild type strains had a similar growth rate in LB broth.

### Uptake of wild type and mutant *Salmonella* strains by transfected U937 cells

**Figure 3:**
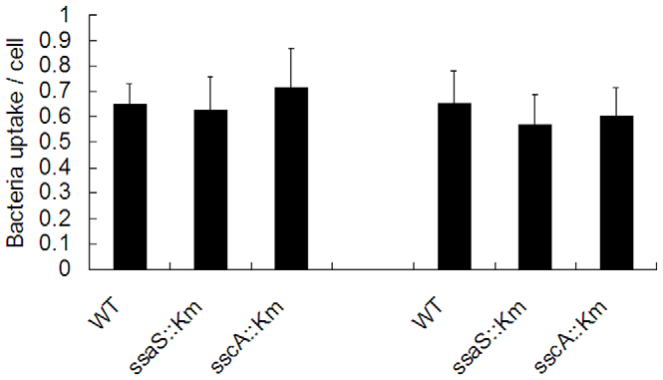
Uptake of *S.* Enteritidis wild type and mutant strains into the transfected U937 cells. Wild type, *ssaS*::Km or *sscA*::Km mutant were used to infect HLA-B27-transfected cells and HLA-A2-transfected cells. The numbers of the bacteria were counted at 1 hour post-infection. The data represented the mean and standard deviation of at least three independent experiments with duplicate samples. No difference was found in the uptake of mutants and wild type bacteria in HLA-B27 cells, and no difference was found in the uptake of mutants and wild type bacteria between HLA-B27 and HLA-A2 cells.

HLA-B27- or HLA-A2-transfected U937 cells were infected with the mutants and wild type bacteria, and uptake of the bacterial strains by U937 cells was measured at 1 hour after infection. The number of living bacteria per cell was counted after lysis of host cells. As shown in Fig. 3, there was no difference in the uptake of mutants and wild type bacteria in HLA-B27 cells, indicating that mutations on these SPI-2 genes did not influence uptake of *Salmonella* into host cells. There was no difference in the uptake of mutants and wild type bacteria between HLA-B27 and HLA-A2 cells either (Fig. 3).

### Enhanced intracellular replication of mutants in U937 cells

**Figure 4:**
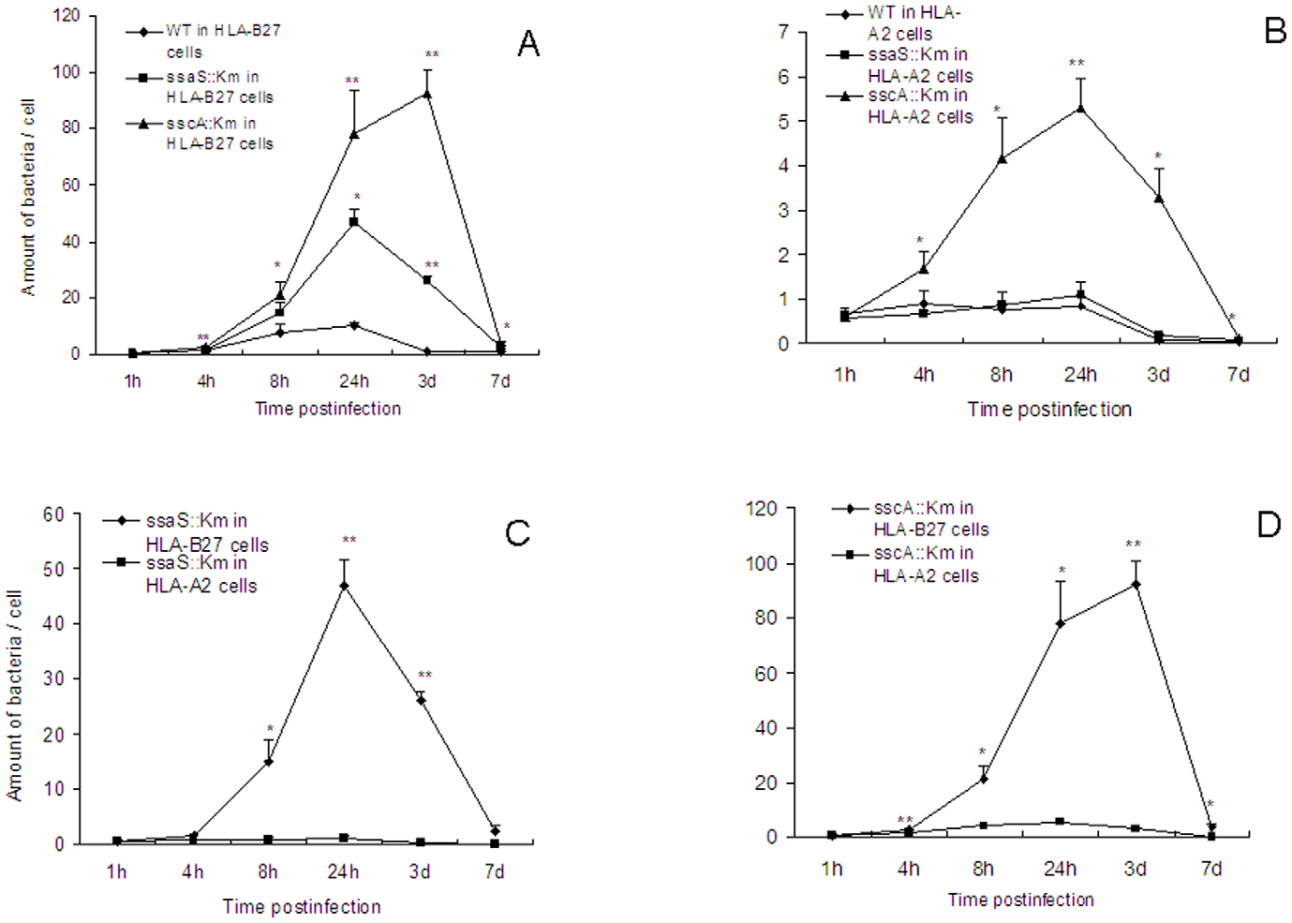
Intracellular growth of wild type and mutant strains in U937 cells. The growth of wild type, the *ssaS*::Km and *sscA*::Km mutants were compared in HLA-B27-transfected (A) or HLA-A2-transfected U937 cells (B). The comparison for the mutant *ssaS*::Km growth in HLA-B27 and HLA-A2 cells (C) and for the mutant *sscA*::Km growth in HLA-B27 and HLA-A2 cells (D) was shown. Values represented the mean and standard deviation of at least three independent experiments with duplicate samples. *, ** and *** indicate *P*<0.05, <0.01 and <0.001, respectively, when the mutant-infected cells compared to the wild type (WT)-infected cells (A and B) or the mutant-infected HLA-B27 cells to HLA-A2 cells (C and D). Data were compared using Student's paired 2-tailed *t*-test.

The cells infected with wild type and mutant *Salmonella* strains were incubated for indicated time points and the numbers of intracellular bacteria were determined. To our surprise, intracellular replication of the two mutant strains in U937 cells was increased compared to wild type strain (Fig. 4A and B). Intracellular replication of both mutants was significantly enhanced in HLA-B27 cells compared to in HLA-A2 cells (Fig. 4C and D). Among the two mutants, the number of the *sscA*::Km mutant bacteria was higher than that of the *ssaS*::Km mutant, and the highest amount of bacteria was detected at 3 days after infection. The increased number of mutated bacteria in infected HLA-B27-transfected U937 cells must be due to enhanced replication since no difference was seen in uptake of mutants and wild type bacteria (Fig. 3). The results suggest that the two SPI-2 genes in some way suppressed intracellular growth in wild type *Salmonella*. It seems that *sscA* had more negative effect on *Salmonella* intracellular growth than *ssaS* did. The two mutants had a small growth advantage when grown in HLA-A2 cells compared to the wild type bacteria. This growth advantage in HLA-A2 cells was much less than in HLA-B27 cells. These results indicate that HLA-B27 significantly modulates *Salmonella* intracellular replication.

### Increased production of TNF-α in HLA-B27-transfected cells induced by the mutants

**Figure 5:**
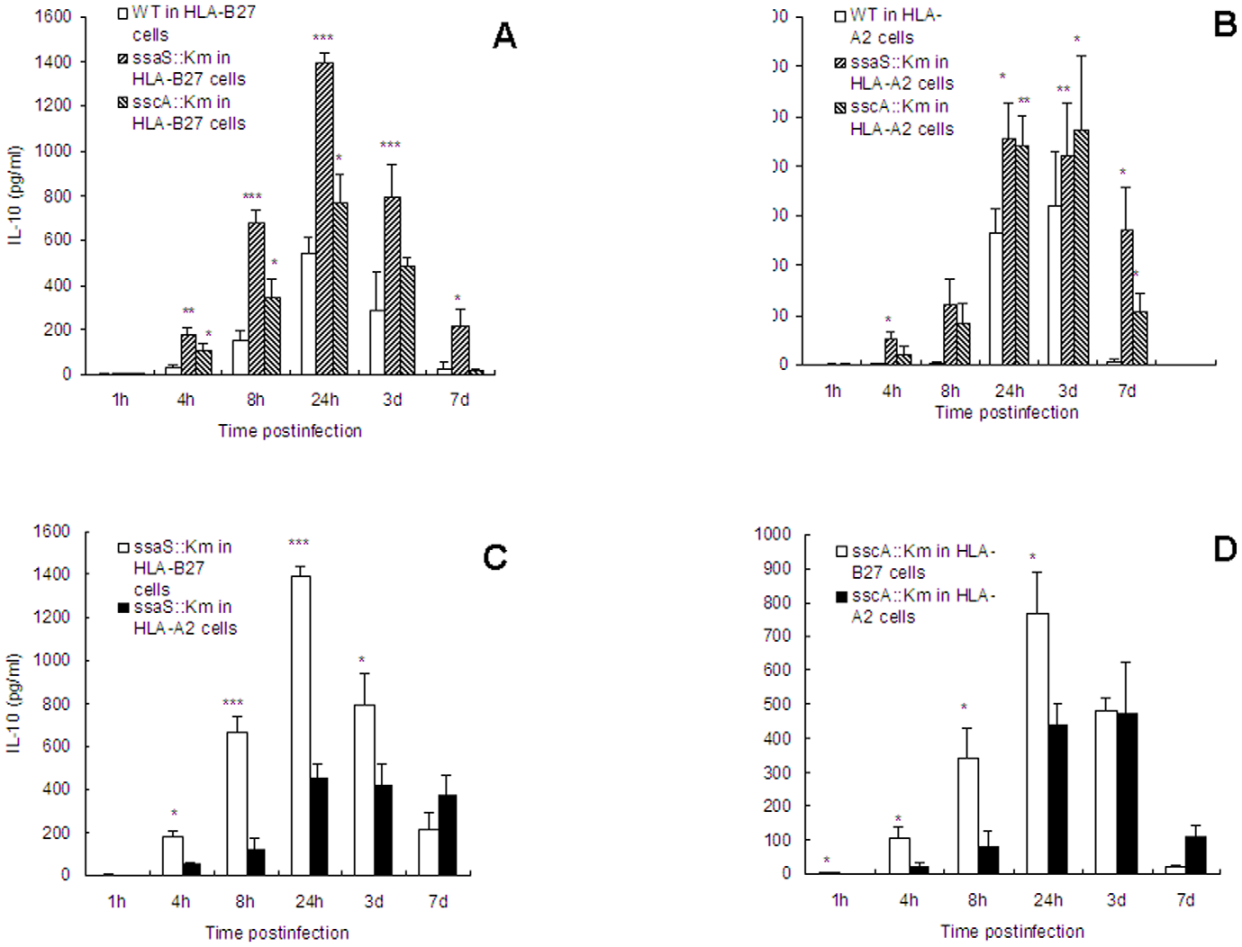
TNF-α production in culture supernatants of U937 cells infected with wild type and mutant strains. Culture supernatants were collected at the indicated time points after infection, the production of TNF-α induced by the wild type, *ssaS*::Km and *sscA*::Km mutants were determined in HLA-B27-transfected U937 cells (A) and in HLA-A2-transfected U937 cells (B). The comparison of TNF-α production induced by the mutant *ssaS*::Km in HLA-B27 and HLA-A2 cells (C) and by the mutant *sscA*::Km in HLA-B27 and HLA-A2 cells (D) was shown. The results represented means ± standard deviation from at least three independent experiments. *, ** and *** indicate *P*<0.05, <0.01 and <0.001, respectively, when the mutant-infected cells compared to the wild type (WT)-infected cells (A and B) or the mutant-infected HLA-B27 cells to HLA-A2 cells (C and D). Data were compared using Student's paired 2-tailed *t*-test.

To investigate increased replication of bacteria in HLA-B27 cells, secretion of TNF-α in culture supernatants was detected using ELISA method. The results indicate that TNF-α was rapidly induced in *Salmonella*-infected U937 cells starting at 1 hour after infection, and maximal production was reached at 8 h after infection with the two mutants (Fig. 5A and B). Compared to HLA-A2 cells, the two mutants caused significantly increased production of TNF-α in HLA-B27 cells (Fig. 5C and D). Moreover, the concentration of TNF-α induced by *ssaS*::Km mutants in HLA-B27 positive cells was at least 2-fold higher than that by the wild type strain, while the mutant *sscA*::Km caused only slightly increased amount of TNF-α. Taken together, these findings suggest that the production of TNF-α is related to the presence of HLA-B27 antigen and *Salmonella* intracellular growth in infected HLA-B27 cells.

### Increased production of IL-10 in HLA-B27-transfected cells by the mutants

**Figure 6:**
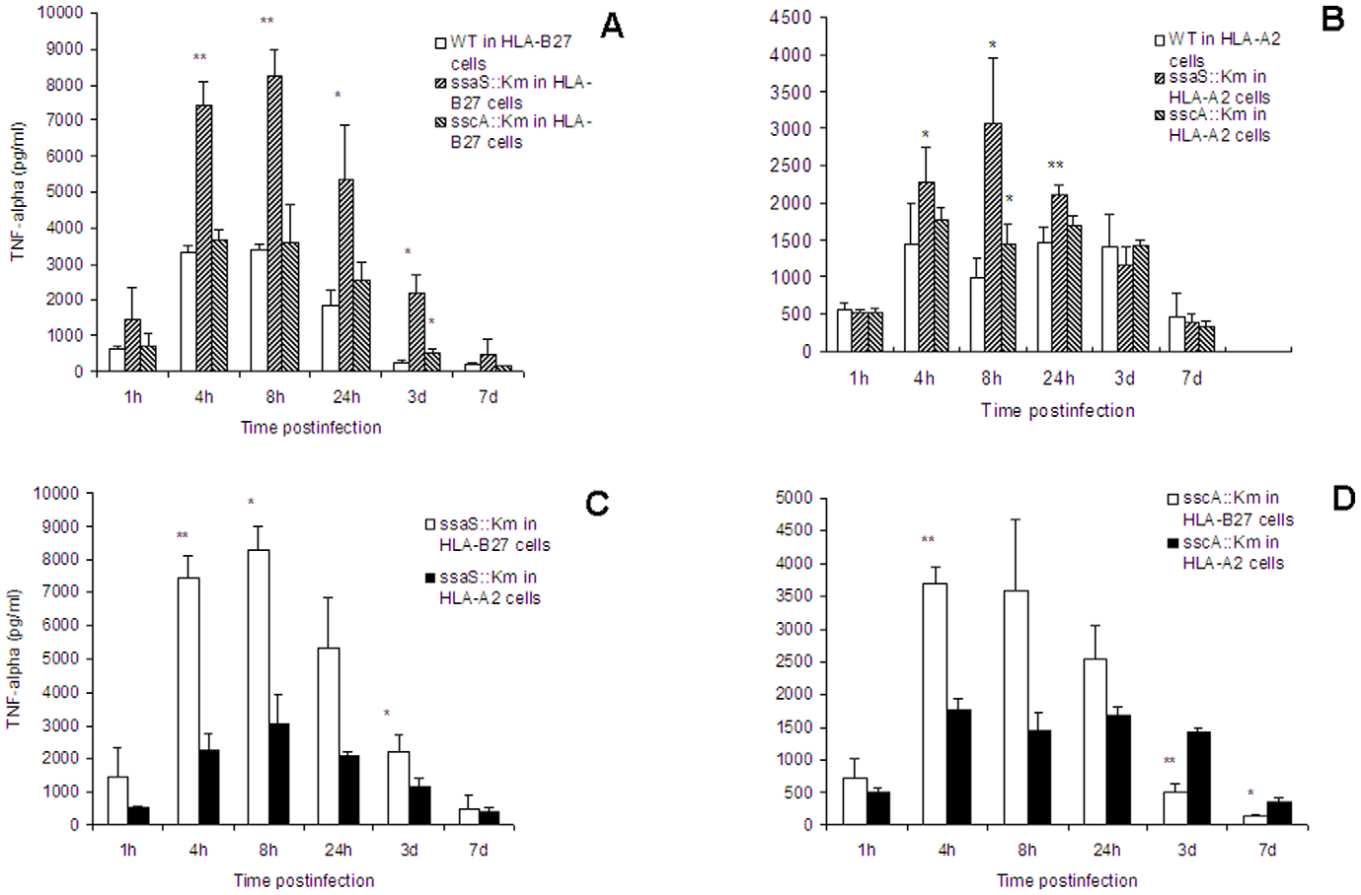
IL-10 production in culture supernatants of U937 cells infected with wild type and mutants. Culture supernatants were collected at the indicated time points after infection, the production of IL-10 induced by the wild type, *ssaS*::Km and *sscA*::Km were determined in HLA-B27-transfected U937 cells (A) and in HLA-A2-transfected U937 cells (B).The comparison of IL-10 production induced by the mutant *ssaS*::Km in HLA-B27 and HLA-A2 cells (C) and by the mutant *sscA*::Km in HLA-B27 and HLA-A2 cells (D) was shown. The results represented means ± standard deviation from at least three independent experiments. *, ** and *** indicate *P*<0.05, <0.01 and <0.001, respectively, when the mutant-infected cells compared to the wild type (WT)-infected cells (A and B) or the mutant-infected HLA-B27 cells to HLA-A2 cells (C and D). Data were compared using Student's paired 2-tailed *t*-test.

IL-10 was detectable at 4 hours after infection and maximal production occurred at 24 h after infection. The mutants produced more IL-10 in both cell lines than the wild type bacteria (Fig. 6A and B). Mutant *ssaS*::Km-infected HLA-B27 cells produced over 2-fold amount of IL-10 compared with the wild-type–infected cells, whereas mutant *sscA*::Km-infected HLA-B27 cells showed a moderate increase in IL-10 production. In addition, the mutants caused more production of IL-10 in HLA-B27 cells than in HLA-A2 cells (Fig. 6C and D), indicating that secretion of IL-10 was also affected by HLA-B27 during *Salmonella* infection.

## Discussion

The present study demonstrates that intracellular replication of the two SPI-2 mutants of *S*. Enteritidis was remarkably increased compared to the replication of wild-type bacteria in HLA-B27 positive human U937 monocytic cells. The results suggest that intracellular growth of wild type bacteria was suppressed in HLA-B27 cells. The observations provide the evidence for the new concept that certain pathogens limit their intracellular growth to keep the balance between the growth and persistence. The two *Salmonella* SPI-2 mutants were significantly increased mainly in HLA-B27 cells compared to in HLA-A2 cells (Fig. 4), suggesting that HLA-B27 antigen plays a key role. Previously, several different theories were proposed to explain the role of HLA-B27 in the pathogenesis of ReA [4], [34]. Our earlier studies showed evidence that HLA-B27 influences the intracellular persistence of arthritis-triggering *Salmonella* within monocytes [6], [10]. These results support the hypothesis of ineffective elimination of microbes in the pathogenesis of ReA [35]. ReA-triggering bacteria may persist for an abnormally long time in HLA-B27-positive patients with ReA [36]. Nevertheless, the interplay between genetic predisposition of HLA-B27 and *Salmonella* genes could lead to the explanation on the increased intracellular growth and long persistence of the pathogens in the HLA-B27 positive patients with ReA.

HLA-B27 probably modulates intracellular environment where bacteria reside which then affects bacterial gene expression. Our previous investigation revealed that 118 *S*. Enteritidis PT4 KS8822/88 genes were up- or down-regulated by HLA-B27 antigen during infection of U937 cells. Among regulated genes SPI-2 genes were up-regulated most [11]. The virulence genes clustered in SPI-2 are necessary for *Salmonella* intracellular growth [37]. The present study demonstrates that the two SPI-2 mutants remarkably increased intracellular growth when compared with that of the wild type *Salmonella* in HLA-B27-transfected macrophages. This highly enhanced growth of mutants was caused by HLA-B27 since only slight difference in the growth rate of the bacteria in HLA-A2 positive cells (Fig. 2) was observed. This gene “function-gain mutation” has been found also in other bacteria. Zahrt and Deretic reported that the growth of the gene *Rv0981*::Km^r^ mutant was significantly higher than the growth of wild-type *Mycobacteria tuberculosis* H37Rv after infection of murine macrophage-like J774 cells and bone marrow-derived macrophage cells, but the mutants were attenuated for virulence [38]. These results were against the gene “function-loss mutation” concept. This phenomenon might reflect the balance between disease-causing bacteria and their persistence during bacterial pathogenesis [39].

TNF-α is considered an important cytokine in the development of arthritis [16]. Anti-TNF-α therapies have been effective in the treatment of SpA, indicating that TNF-α plays a major role in the pathogenesis of SpA [13], [14]. The central role of TNF-α was further supported by genetic study that TNFRp55-deficient mice were highly susceptible to *Salmonella* infection [18]. The present study demonstrated that SPI-2 mutants induced higher production of TNF-α than wild-type bacteria in infected HLA-B27-transfected cells, suggesting that the production of TNF-α may be associated with the growth of *Salmonella* in HLA-B27-transfected U937 cells. On the other hand, both the amount of bacteria and production of TNF-α were obviously enhanced by HLA-B27, indicating that HLA-B27 has a regulatory role in the *Salmonella* replication and secretion of certain cytokines intracellularly. Gamma interferon (IFN-γ) is a major activator of macrophages and the role of IFN-γ in natural resistance to bacteria in mice and host cells has been documented [40]. TNF-α and IFN-γ appear to cooperate in the activation of macrophages [41]. However, no IFN-γ was detectable from either HLA-B27-transfected cells or HLA-A2-transfected cells (data not shown).

IL-10, one of inhibitory cytokines, generally deactivates macrophages and permits enhanced bacterial intracellular growth during infection [21], [42]. This study shows that SPI-2 mutants induced more IL-10 than did the wild type, correlating to increased number of mutant bacteria in HLA-B27 cells. The SPI-2 mutants caused a moderate increase of IL-10 in HLA-A2 cells, but not so much as in HLA-B27 cells (Fig. 6), suggesting that HLA-B27 regulated IL-10 secretion. We showed earlier that HLA-B27-tranfected cells produced more IL-10 during the first three days postinfection than HLA-A2-transfected cells which was correlated with increased bacterial survival in the HLA-B27 cells [8]. However, the administration of neutralizing mAb against IL-10 did not notably affect the clearance of the bacteria. This may be explained by the perhaps incomplete elimination of endogenous IL-10 due to the addition of anti-IL-10 being not enough [8].

HLA-B27 modulates interaction between host and *Salmonella* during infection [43]. This interaction involves the exchange of biochemical signals, called “cross–talk” [44]. As a result of this talk, *Salmonella* translocates a number of effector proteins into host cells via type III secretion systems to elicit host cell's signaling pathway, leading to a variety of host response, including induction of cytokines [45]. Uchiya et al [21] showed SPI-2 gene *spiC* (*ssaB*) to be involved in the protein kinase A (PKA) signaling pathway that induces IL-10 expression in *Salmonella*-infected macrophages. This played an important role in intracellular growth of *Salmonella*. In this study, we showed that SPI-2 genes suppress secretion of IL-10 and bacterial intracellular replication, and the extent of suppression is augmented by HLA-B27. Collectively, these observations indicate that SPI-2 genes trigger host cell signaling transduction pathways to moderate production of cytokines and affect intracellular growth.

In conclusion, we demonstrated enhanced intracellular growth of two SPI-2 mutants of *Salmonella* within HLA-B27-transfected U937 cells. The observations suggest that SPI-2 genes in wild-type *Salmonella* somehow suppress intracellular growth and secretion of cytokines TNF-α and IL-10. This study provides evidence that wild-type *Salmonella* limits its intracellular growth in the HLA-B27-transfected cells to keep the balance between its growth and pathogenesis. However,further experiments will be still required to determine how *Salmonella* SPI-2 genes affect intracellular signalling pathways and modify *Salmonella* replication in HLA-B27 positive cells.

## Materials and Methods

### Cell lines and transfection

The human monocytic cell line U937 was obtained from American Type Culture Collection (Rockville, USA). The U937 cell line expresses the HLA class I alleles A3, A26, B18, B51, Cw1, and Cw3 [46]. Co-transfection of the cells with the vectors harbouring HLA-B*2705 or HLA-A2 genomic DNA with the plasmid pSV2neo (to confer resistance to geneticin [G-418]) was carried out by electroporation. The cells were cultured in RPMI 1640 medium supplemented with 10% heat-inactivated fetal calf serum (FCS; PAA laboratories, Linz, Austria), 1.8 mM L-glutamine and 50 µg/ml gentamicin (both from Biological Industries, Kibbutz Beit Herennek, Israel) at 37°C in a humidified atmosphere of 5% CO_2_/95% air incubator. The expression of transfected HLA-B27 or HLA-A2 molecules on the surface of the U937 cells was examinated by FACScan flow cytometry (BD Immunocytometry Systems, San Jose, CA) each time when the new batch of cells was thawed for use. Cells were stained with fluorescein isothiocyanate-conjugated anti-human HLA-B27 monoclonal antibody (mAb) (clone FD705-9EIEI0; One Lambda, Canoga Park, CA) and anti-HLA-A2 monoclonal antibody (mAb) (Clone BB7.2; One Lambda Inc., Canoga Park, CA) as described previously [6].

### Bacterial strains, plasmids and culture conditions

The wild type *Salmonella* Enteritidis strain was originally isolated from a stool of a patient with *Salmonella*-triggered ReA [6], typed as phage type 4 (PT4) and named as *Salmonella* Enteritidis PT4 KS8822/88 [11]. The mutants from *Salmonella* wild type were made as described below. *Escherichia coli* carrying temperature-sensitive plasmid (pKOBEGA) or MC4100 ybeW:Km were gifts from Dr. Ghigo. The *Salmonella* and *E. coli* strains were routinely grown in Luria-Bertani (LB) broth or on LB agar plates at 37°C or as indicated. Media were supplemented with ampicillin (100 µg/ml) and kanamycin (30 µg/ml) (both from Sigma) as required.

### Infection of cells with *S.* Enteritidis PT4 KS8822/88

The U937 cells were diluted to a concentration of 1.0×10^6^ cells/ml and seeded in tissue culture flasks (75-cm^2^) or 24-well plates (Greiner, Germany). For differentiation to adherent macrophages, the cells were incubated with 10 ng/ml phorbol myristate acetate (PMA; Sigma-Aldrich, St Louis, MO) for 24 hours. Meanwhile, *Salmonella* strains were grown overnight in LB broth to the logarithmic phase of growth. Then *Salmonella* bacteria were used to infect the PMA -stimulated U937 cells at a ratio of 50∶1 (bacteria∶cell) without antibiotics for 1 h at 37°C [11]. Then the infected cells were washed three times to remove non-adherent bacteria and overlaid with fresh 1640 medium containing gentamycin (50 µg/ml) to kill the remaining extracellular bacteria. The *Salmonella*-infected cells were then incubated at 37°C for 1, 4, 8, 24 h, 3 or 7 d as indicated. To determine the number of living intracellular bacteria, the infected cells were scraped using a cell scraper and the amount of living host cells was counted under a microscope after staining with Trypan blue. Host cells were lysed with 1% Triton X-100 in 1× phosphate-buffered saline (PBS) at room temperature for 5–10 min, 20 µl of the lysate in ten-fold serial dilutions in PBS were added to LB agar plates, and the numbers of bacteria were reported as CFU (colony forming units) [6].

### Mutagenesis of *S.* Enteritidis PT4 KS8822/88

Mutagenesis of *Salmonella* SPI-2 genes *ssaS* and *sscA* was carried out using a one-step inactivation method first described in *E. coli* by Datsenko and Wanner [47] and developed for *Salmonella* strain by Solano et al [48] with some modifications in this study. The hybrid oligonucleotide primers (Table 1) contained the priming sequences to amplify kanamycin resistance gene using *E. coli* MC 4100 ybeW:Km genomic as template and homology extensions for homologous recombination. The homology extensions were from the nucleotide +22 to +81 and +115 to +174 within the internal region of *ssaS* gene, or the nucleotide +187 to +246 and +317 to +376 within that of *sscA* gene. The resulting PCR products containing homology extension and kanamycin cassette was used to transform wild type *Salmonella* strain carrying helper plasmid pKOBEGA, which encodes λ Red recombinase and promotes allelic exchange between the genomic DNA and the PCR product. After transformation by electroporation (25 µF, 200 Ω, 2.5 kV), transfectants were grown in LB plates with 30 µg/ml kanamycin. The selected transfectants (mutants) were tested for loss of the appropriate region of DNA sequences by PCR amplification. The total genomic DNA from mutant and wild type *Salmonella* strains was isolated using High Pure PCR template Preparation kit (Roche) and then used as template. Using flanking primers (Table 1), the PCR amplification was performed to confirm the deletion of DNA sequences in mutant strains. Subsequently, the mutants were grown in LB broth at 43°C to cure the helper plasmid.

### Measurement of cytokines in cell-culture supernatants of infected cells

The cytokines were measured using enzyme-linked immunosorbent assay (ELISA). Supernatants were collected from 24-well plates at the defined time points: 1, 4, 8, 24, 72 h, 3 or 7 d after infection. The assay was performed in 96-well plates (Nunc) with capture anti-human antibodies. The antibody pairs: mAb1 and mAbm11 for measurement of TNF-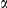; and JES3-9D7 and JES3-12G8 for IL-10 were purchased from Pharmingen (San Diego, California). The absorbances were measured with a Victor™ Multilabel Counter (Wallac Oy, Turku, Finland) at a wavelength of 405 nm. Concentrations were calculated from a standard curve generated from each plate.

### Statistical analysis

Differences in cytokine levels in cell cultures as well as intracellular growth of wild type and mutant strains were analysed using unpaired two-tailed Student's t test. *P* values of 0.05 or less were considered significant.
